# The Brief Form of the Test of Gross Motor Development-3 for Individuals with Visual Impairments

**DOI:** 10.3390/ijerph18157962

**Published:** 2021-07-28

**Authors:** Ali S. Brian, Angela Starrett, Adam Pennell, Pamela Haibach Beach, Sally Taunton Miedema, Alexandra Stribing, Emily Gilbert, Matthew Patey, Lauren J. Lieberman

**Affiliations:** 1 Department of Physical Education, University of South Carolina, Columbia, SC 29208, USA; staunton@email.sc.edu (S.T.M.); stribing@email.sc.edu (A.S.); 2 Child Development Research Center, University of South Carolina, Columbia, SC 29208, USA; starrett@mailbox.sc.edu; 3 Natural Science Division, Pepperdine University, Malibu, CA 90263, USA; adam.pennell@pepperdine.edu; 4 SUNY-Brockport, Kinesiology, Sport Studies, and Physical Education, Brockport, NY 14420, USA; pbeach@brockport.edu (P.H.B.); llieberman@brockport.edu (L.J.L.); 5 SUNY-Cortland, Physical Education Department, Cortland, NY 13045, USA; emily.gilbert@cortland.edu; 6 Department of Movement Arts, Health Promotion, and Leisure Studies, Bridgewater State University, Bridgewater, MA 02324, USA; mpatey@bridgew.edu

**Keywords:** motor competence, fundamental movement skills, assessment, psychometrics, blindness

## Abstract

Children with visual impairments (VI) tend to struggle with their fundamental motor skills (FMS), and these difficulties often persist across the lifespan, requiring frequent assessment. The Test of Gross Motor Development (TGMD) shows robust psychometric properties for children with VI. The TGMD, which includes 13 skills, is time-consuming to administer and score, warranting the need to explore brief versions. Therefore, the purpose of this study was to explore the psychometric properties of three, six-skill versions of the TGMD-3 with children with VI. Children (*n =* 302; Boys = 58%, Girls = 42%; Mage *=* 13.00, SD = 2.50 years) with VI (B1 = 27%, B2 = 20%, B3 = 38%, B4 = 15%) participated in this study. We examined three different models using confirmatory factor analyses on the relationships between the motor skills and latent traits across the models. Scores from all three brief versions had acceptable global fit. Although further research should be conducted, practitioners can adopt a brief version of the TGMD to assess children with VI.

## 1. The Brief Form of the Test of Gross Motor Development-3 for Individuals with Visual Impairments

Fundamental motor skills are a subset of gross motor skills (body movement patterns which are goal-directed) that includes manipulating objects (e.g., ball, object control, or manipulative skills—throw, catch, strike), keeping the body “stabilized” (e.g., twisting, bending, curling), or moving the body from one point in space to another (e.g., locomotor skills—run, jump, hop) [1]. Fundamental motor skills are the “building blocks” to more complex movements necessary to play sports, games, or participate in physical activity behaviors [1]. Thus, it is no surprise that fundamental motor skill in childhood/adolescence can predict physical activity behaviors throughout the lifespan [2].

Unfortunately, children, adolescents, and youth (herein referred to as children) with visual impairments (VI), including blindness, tend to struggle with their fundamental motor skills, and these difficulties often persist to the extent of arrested/delayed development across the lifespan [3,4]. However, if children with VI receive intervention, their fundamental motor skill difficulties can resolve quickly [3] and to the level needed to support health-enhancing levels of fitness [5]. Thus, it is critical that children with VI have their fundamental motor skills assessed as early and as frequently as possible.

The Test of Gross Motor Development (2nd edition [6] or 3rd edition [7]) is one of the most widely adopted assessments of fundamental motor skill in the United States and across the world [8,9] and its psychometric properties were specifically vetted for children with VI [10]. The TGMD is not only a common choice of practitioners for assessing children with VI in rehabilitation [11], but also for researchers exploring descriptive, cross-sectional, and/or longitudinal inquiries [3,4,5].

Despite its popularity among practitioners and researchers, there are some challenges to both administering and scoring the TGMD (2nd or 3rd editions) with large samples and in school-based settings due to the time needed to administer and score [8,9,12]. Administration of the TGMD-3 requires 20–30 min per child. Scoring the TGMD-3 may require an additional 40–60 min per child [6,7]. Thus, it is not surprising that time tends to be the more frequently reported barrier for assessing gross motor skills among teachers [12].

For practitioners, time with their students (if teachers) or patients (if employed in rehabilitative settings) is quite limited. Often, teachers and physical therapists see their students/patients 30 min a week for physical education or for therapy sessions. Faster assessments in terms of administration and scoring may increase likelihood of assessment amongst practitioners. More frequent assessment often leads to improved downstream effects for children with VI (e.g., lessons rooted in present level of performance, knowledge of program effectiveness, etc. [11,13]).

For researchers, time can also be of the essence when assessing children with VI as access to the population tends to occur via schools or short-run events (e.g., camps). Visual impairment is considered low incidence among children as there are approximately 547,000 children with VI (including those with vision difficulty despite the use of corrective lens) in the United States [14] of which approximately 68,000 are considered legally blind [15]. As a result, research teams need to travel to many schools or sites to accumulate a large enough sample sufficiently powered for analyses. Furthermore, schools and short-run events tend to provide very limited time for research. Thus, it is very difficult and expensive to capture robust, powered data sets given the time constraints inherent within standardized motor assessments with good psychometric properties. Furthermore, fewer skills should be faster to both administer and score (e.g., half as many items should cut time in half, addressing the time constraints above), warranting the need to explore the extent to which a brief version of the TGMD-3 can retain the stout psychometric properties of the full battery for use with children with VI.

Along with time constraints, the use of the TGMD-3 for children with VI might oblige criticism regarding its ecological and external validity. Ecological and external validity are somewhat conflated terms but are not synonyms. Ecological validity refers to the extent to which research occurs in a setting that best mimics the real world [16]. Similarly, external validity refers to the extent to which findings from a study can then generalize from that specific sample to a larger population. Thus, studies with good external validity may not be ecologically valid and vice versa; however, assessing outcomes in a manner that best mimics “real world” settings can certainly improve external validity [16].

For children with VI, many previous studies suffer from issues with external validity as samples are often limited in size and occur in somewhat contrived settings (e.g., laboratories; [17]). Issues with ecological validity also emerge as many skills within test batteries may not be familiar [18], may not be developmentally appropriate (e.g., should an 18-year-old gallop), or may not translate to the sports and games specifically designed for children/adults with VI (e.g., there is no throwing in Beep Baseball). Completing the entire test battery (e.g., 13 skills on the TGMD-3) may result in issues surrounding ecological validity. For example, certain items within the battery may be more appropriate than others depending on the age, location, and culture of the sample. In addition, older samples of children (e.g., 11 years+) with VI may participate in the United States Association for Blind Athletes (USABA) track and field competition. Thus, hopping and running are appropriate, familiar, and relevant. Furthermore, Beep Baseball is one of the most popular sports in the United States for children and adults with VI, supporting the need to assess strike, catch, and run. In other countries, other Paralympic sports are more the norm. For example, children with VI in Brazil may be more likely to participate in 5-a-side soccer (a new/growing sport in the United States) or goalball (Paralympic sport) warranting the assessment of kick and roll (or underarm throw). Thus, brief versions of the TGMD-3 for children with VI that include different skills warrant exploration.

## 2. Potential/Proposed Brief TGMD-3 Batteries for Children with VI

The TGMD-3 is a two-factor model (locomotor and ball skill subscales) with psychometric properties that are typically stout [7]. While developed for children without disabilities, Brian and others [10] showed that the two-factor structure holds for children with VI. According to Kline [19], any factor within a model should have at least three items. Therefore, target/exemplar models had to have at least three skills per factor, and we followed that suggestion in the following ways.

### 2.1. Brazilian TGMD-2 Short Form (TGMD-2-SF; V-6)

There is precedent for adopting a brief version of the TGMD-2 [14,20]. Although originating from the TGMD-2 (and not the current TGMD-3), Valentini and colleagues [21] introduced a short form for the TGMD-2 using Brazilian youth aged three to ten years of age. Using exploratory and confirmatory factor analyses, the two-factor TGMD-2-SF included the run, gallop, and hop (i.e., locomotor factor) and the two-handed strike, kick, and throw (i.e., object control/ball skill factor) skills. Due to the previously established precedence of this short form (albeit in Brazilian youth without VI using a previous version of the TGMD), the skills within the TGMD-2-SF were investigated as a potential two-factor solution within the current sample (referred to as V-6).

### 2.2. A Novel, Paralympic, and VI Sport-Specific Inspired Short Form (SPT-6)

Based on sports that are common in the VI community (e.g., goalball, beep baseball, blind 5-a-side soccer) and the foundational requirements needed for skilled movement/mobility (e.g., strength, power, rhythm, coordination) the run, hop, and skip (locomotor factor) as well as the two-hand strike, kick, and underhand throw (ball skill factor) were hypothesized skills for the proposed VI sport-specific battery. For example, the two-hand strike is a significant component of beep baseball (i.e., batting) while the underhand throw (also described as the toss) is biomechanically similar to rolling as rolling is a major component of goalball. Unfortunately, although present in the TGMD-2, the roll was removed from the TGMD-3 [6,7]. The kick was included as it is a necessary skill in 5-a-side soccer and, importantly, the kick represents lower-body ball skill motor competence. Concerning locomotor skills, running is a ubiquitous transportation skill. The hop requires a significant amount of power production and dynamic postural control while both the hop and the skip require substantial inter-limb coordination and rhythmicity. While rationales were provided for the inclusion of each skill, youth with VI may not regularly participate in and/or have consistent access to the acknowledged VI-specific sports. Thus, while the selection of said VI-specific skills was advisable, the premise that such skills may be developed, experienced, or were motorically representative of American youth with VI was based on ecological assumptions/hypotheses.

### 2.3. Previous Results of Psychometrics of TGMD-3 for Children with VI (STAT-6)

In order to determine a statistically based brief form, we selected six skills based upon the factor loadings from the original psychometric evaluation of the TGMD-3 for children with VI [10]. Using factor loadings, the top three ball skills (i.e., overarm throw, dribble, and one-hand strike) and two of the top three locomotor skills (i.e., run and hop) were included. We chose to not include skip as it was already the third locomotor skill in the SPT-6 model. As such, we strategically opted for slide instead of jump since the slide shows discrimination into the frontal plane and out of the sagittal plane. Most children with VI are taught to move within the sagittal plane from orientation and mobility specialists by using their canes [22]. However, the ability to locomote within the frontal plane may better predict the ability to avoid obstacles and shed light into co-morbidities associated with VI that result from cortical or cerebral etiologies [23]. Thus, we wanted to assess movements into the frontal plane in concert with skills that assess movements within the sagittal plane (e.g., running).

Although there are previous inquiries to support the statistical and theoretical basis of this study, it is important to explore the psychometric properties of brief versions of the TGMD-3 specifically for children with VI. Thus, the purpose of the current study was to examine the psychometric properties of the three brief versions of the TGMD-3 for adequately measuring locomotor and ball skills in children with VI. We hypothesize that the STAT-6 and SPT-6 models, developed for US children with VI statistically and contextually, respectively, will measure locomotor and ball skills as well as the full TGMD-3 battery. We were unsure how the V-6 model would perform compared to the full battery, as this brief version was previously vetted on Brazilian children without VI.

## 3. Methods and Materials

### 3.1. Design, Participants, and Setting

This study featured a descriptive-analytic design with convenience sampling. Children (*n =* 302; Boys = 58%, Girls = 42%; Mage *=* 13.00, SD = 2.50 years) with VI (B1 = 27%, B2 = 20%, B3 = 38%, B4 = 15%; see below for definitions) participated in this study. This study occurred at several seven-day sports camps specifically designed for children with VI across New York, Florida, and Texas. All children with VI who attended these camps were invited to participate.

### 3.2. Instrumentation

#### 3.2.1. United States Association of Blind Athletes (USABA) Classification System

Participants were stratified across degree of vision based upon the United States Association for Blind Athletes (USABA) classification scale. The USABA scale includes four levels: B1 includes no light perception in either eye up to light perception, as well as an inability to recognize the shape of a hand at any distance or in any direction and is considered the lowest level of visual acuity. Children who are B2 can recognize the shape of a hand with a visual acuity up to 20/600; B3 includes 20/600–20/200. Both B2 and B3 may or may not include a visual field of less than 5° in the best eye including eye correction. In contrast, children who are B4 possess visual acuity above 20/200 and up to 20/70, as well as a visual field larger than 20° in the best eye with the best practical eye correction.

#### 3.2.2. TGMD-3

The TGMD-3 [24,25] includes 13 items within two subscales: locomotor and ball skills. The locomotor subscale contains six items (run, jump, hop, skip, gallop, and slide) and the ball skills subscale includes seven object control skills (catch, kick, strike with a bat, strike with a racquet, underarm throw, overhand throw, and dribble) that are each scored on several performance criteria, resulting in a raw score ranging between 0–100 (locomotor = 46, ball skills = 54) points. Standardized procedures include: (1) a verbal and visual demonstration for each skill one at a time; (2) one practice trial for each skill (again, one skill at a time); (3) if necessary, the repetition of an additional verbal and visual demonstration after the practice trial; and (4) two trials (by the participants) without cueing or assistance from the administrator. All trials should be digitally video recorded for scoring purposes [7]. The TGMD-3 holds good psychometric properties for youth without VI ages 3 to 10 years, 11 months [7].

In 2018, Brian and colleagues [10] established the psychometric properties of the TGMD-3 for children/adolescents (ages 8–19 years) with VI. The psychometric properties were quite stout, revealing high internal consistency (ω = 0.89–0.95), strong interrater reliability (ICC = 0.91–0.92), convergence with the TGMD-2 (*r =* 0.96), and good model fit, *χ*^2^(63) = 80.10, *p =* 0.072, χ^2^/df ratio = 1.27, RMSEA = 0.06, CFI = 0.97. Not only were the psychometrics acceptable, but Brian and colleagues also established recommendations to modify the TGMD-3 for individuals with VI as needed [10]. Modifications included: (1) using least-to-most prompting for the demonstration (no modifications, demonstration from different angles, tactile modeling, the use of verbal cues only if necessary); (2) placing audio devices, such as a buzzer, or a human guide as a clapper at both the start/stop points for each locomotor skill item (only if needed); (3) using bright-colored/reflective tape around the boundaries; and (4) allowing the participant to walk the test area, if necessary, to assure their safety for comfort [10]. Historically, there have been no significant differences when those without VI performed the TGMD with and without the same affordances/modifications for those with VI [24]. Furthermore, it is acceptable to administer the TGMD-3, using raw scores for analyses, when participants fail to reach a maximum score, if they are beyond the age of 11 years [25].

### 3.3. Procedures

The Institutional Review Board of the University of South Carolina (CR00026556) approved all procedures. Parents of participants provided written informed consent and completed a demographic survey for their children while all child participants provided verbal assent. All participants completed the entire TGMD-3 battery with members of the research staff following the procedures outlined within the TGMD-3 manual [7] and featuring vetted modifications, if necessary [10]. All TGMD-3 trials occurred within a seven-day period in the summer at various sport camp locations in New York, Florida, and Texas. Research staff digitally recorded each trial and then coded from the digital recordings. All raters passed inter-rater reliability requirements of greater than 80% with the lead researcher.

### 3.4. Analytic Methods

Confirmatory factor analysis (CFA) was an appropriate statistical analysis for our study for several reasons. Gross motor skill is represented as two latent traits: locomotor skills and object control skills [10], and CFA can handle the measurement errors [20]. CFA allowed us to examine the relationships between the observed skills and the two latent traits across the different versions of the TGMD proposed. Lastly, given the a priori decision of a two-factor model, the CFA framework provided more information than exploratory factor analysis. As such, CFA was deemed the most methodologically sound approach for addressing the research questions.

All analyses were completed using Mplus version 8.4 statistical software. The maximum likelihood estimation method was used to estimate parameters. In accordance with current methodological research, global model fit was examined using a variety of fit indices [19]. We considered the *p*-value of the model $\chi^{2}$ statistic, where a nonsignificant value indicated good fit between the hypothesized model and the observed variables. Furthermore, the ratio of $\chi^{2}/\mathrm{df}$ should be a relatively small number with most recommending less than three [19]. We also examined the root mean square error of approximation (RMSEA), the standardized root mean square residual (SRMR), and the comparative fit index (CFI). RMSEA values less than 0.08, SRMR values less than 0.05, and CFI values greater than 0.90 are recommended for relatively good evidence of model-to-data fit [26]. We also considered psychometric properties of the manifest indicator variables, including reliability estimates and convergent validity [27,28]. In Mplus, the parameter estimate (loading) divided by the standard error is treated as a z-score, where significant values for all indicators of the factor indicate convergent validity [29]. Lastly, we examined psychometric properties of the latent factors by calculating composite reliability and the variance extracted estimate. Composite reliability reflects the internal consistency of the individual skills that are hypothesized to measure the latent trait (e.g., locomotor skills). The variance extracted estimate for the latent trait indicates the amount of variance that is captured by that factor in relation to the amount of variance that is due to measurement error. Composite reliability should exceed 0.70 [30], and the variance extracted estimate should exceed 0.50 [31]. Finally, we used global fit indices and the psychometric properties of the observed variables and latent factor to compare our proposed versions of the TGMD-3.

## 4. Results

### 4.1. Model Fit

Descriptive statistics for observed skills are shown in Table 1, and correlations among skills are presented in Table 2. Item difficulty represents the proportion of children with VI who got the maximum score for a skill. Difficulty values for each item should range between 0.20 and 0.80 [32]. Item discrimination is a point-biserial correlation and refers to the ability of a skill to differentiate among children with VI based on how well they perform on locomotor and object control skills. The accepted practice is to omit or revise items with indexes below 0.35 [33]. According to Table 3, nearly all models had acceptable global fit. For the model $\chi^{2}$ statistic, the full TGMD-3 ($\chi^{2}$(64) = 79.39, *p* = 0.093), STAT-6 ($\chi^{2}$(8) = 10.45, *p* = 0.24), and V-6 ($\chi^{2}$(8) = 7.44, *p* = 0.49) had nonsignificant values indicating good fit. Conversely, SPT-6 ($\chi^{2}$(8) = 18.07, *p* = 0.02) had a significant value suggesting possible poorer fit. All models had a ratio of $\chi^{2}/\mathrm{df}$ below the suggested cutoff of 3. All models had CFI values above 0.90, and, similarly, they all had SRMR values below 0.05. With RMSEA, the results were mixed with three of the four models showing acceptable fit, meaning an estimate below 0.08 and preferably a 90% confidence interval reflecting as much. The full TGMD-3 (estimate = 0.028, 90% CI [0.00, 0.05]), STAT-6 (estimate = 0.032, 90% CI [0.00, 0.08]), and V-6 (estimate = 0.00, 90% CI [0.00, 0.06]) had good fit based on both the estimate and the confidence interval. SPT-6 (estimate = 0.065, 90% CI [0.02, 0.10]) had RMSEA estimates below 0.08 but probabilities being in range below 90%.

### 4.2. Psychometric Properties of Individual Skills

Table 4 reports information related to the psychometric properties of the measurement models investigated in the current study. For each skill within each model, standardized loadings are presented, along with z-scores for the loadings and item reliabilities. For each model, the group of skills displayed convergent validity since the factor loading statistic for each skill was significant (*p* < 0.001). Regarding reliability at the skill level, skill reliabilities across the models ranged from 0.19 to 0.58 (Mdn = 0.50). Only gallop (Mdn = 0.19) and slide (Mdn = 0.295) had reliabilities below 0.30.

### 4.3. Psychometric Properties of the Latent Trait

Across all models, composite reliabilities exceeded 0.70 for the latent trait of object control skills. For locomotor skills, most models had composite reliabilities that exceeded 0.70, excluding V-6 (0.63). For the variance extracted estimate for object control skills, STAT-6 (0.55) and V-6 (0.53) had variance extracted estimates above the full TGMD-3 (0.50). SPT-6 (0.46) had a variance extracted estimate below the full TGMD-3. For the latent trait of locomotor skills, STAT-6 (0.42) and SPT-6 (0.47) had variance extracted estimates above the full TGMD-3 (0.39). However, V-6 (0.37) had a variance extracted estimate below the full TGMD-3. Lastly, the correlation between locomotor and object control skills was similar across all four models (TGMD-3: 0.945, STAT-6: 0.923, V-6: 0.989, SPT-6: 0.990).

## 5. Discussion

The purpose of this study was to assess the psychometric properties of brief, six-skill versions of the TGMD-3 for children with VI. Within this study, we examined three different models using CFA to examine the relationships between the motor skills and latent traits across the models. Originally, the psychometrics for the TGMD-3 were acceptable with modified equipment for children with VI [10]. The results of this current study are also very promising, like previous findings on a brief version of the TGMD-2 for Brazilian youth [21].

All models had acceptable global fit, indicating much potential and flexibility in developing a brief version of the TGMD. All models also had good SRMR and CFI values; however, the SPT-6 model showed potential issues with the global model fit based upon the 90% confidence interval for RMSEA. With regard to reliability, all three brief versions were above the cutoff of 0.70 for ball skills, and the STAT-6 and SPT-6 models were above the cutoff for locomotor skills. Regarding variance extracted estimates, the STAT-6 and V-6 models do better than the full TGMD-3 model for ball skills. Similarly, the STAT-6 and SPT-6 models do better than the full battery for locomotor skills. In combination, these results demonstrate how locomotor and ball skills need to be strategically selected for the brief versions for children with VI. Contextually, gallop should be used with caution to measure locomotor skills in children with VI. From our results, we glean that gallop is an extremely difficult skill that has lower discrimination for children in the sample, regardless of degree of VI. Gallop is a continuous skill that relies upon rhythmical control and multi-limb coordination. It is very difficult to provide a tactile model that replicates the gallop. Unsurprisingly, gallop had a low factor loading on both the full TGMD-3 and the V-6 models, resulting in lower reliability and variance extracted for the locomotor latent trait for the V-6 model. These data are similar to those from Brian et al. (2018) when they vetted the full TGMD-3 in children with VI. Could the low discrimination of the gallop be due to the need for a visual representation to mimic the demonstration? Interestingly, these low discriminations did not emerge for the skip (another continuous skill which relies upon rhythm). Plausibly, the contralateral nature of the skip along with its even rhythm pattern lends itself better to featuring physical prompting than gallop (ipsilateral pattern with uneven rhythms). The uneven rhythm of the gallop might require visual information whereas the vertical movements in the skip can be felt via haptics alone, rendering it less reliant upon visual information than the gallop. However, future research efforts should explore different ways though which to demonstrate skills that may be more reliant upon vision (e.g., gallop) than closed ball skills (e.g., two-hand strike).

Concurrently, we see that the underhand throw has a lower factor loading than the other ball skills, resulting in a lower variance extracted estimate for the SPT-6 model compared to the full TGMD-3. Item analysis showed that underhand throw is a somewhat difficult skill with the lowest discrimination among the ball skills. In the a priori model development, underhand toss was strategically selected as the closest TGMD-3 skill to rolling a ball (necessary as the roll (from the TGMD-2) was removed from the TGMD-3). The roll (now underarm throw) is an important skill for goalball and other Paralympic sports. Unfortunately, based upon our results, the underhand throw may be more difficult than rolling a ball in the VI context. The underhand throw requires a judgement of distance (e.g., hit a wall 15 feet away). Judging distance requires figure ground and visual motor integration. Although sound sources can assist judgement, clearly more support is needed. However, future research is needed to discern whether additional supports with the underhand toss might improve its discriminant function and whether rolling a ball (instead of toss) might contribute similarly to the latent trait of ball skills in the VI context.

Overall, the authors recommend using the locomotor skills for the STAT-6 (run, hop, slide) and SPT-6 (run, hop, skip) models and the ball skills for the STAT-6 (one-hand strike, dribble, overhand throw) and V-6 (two-handed strike, kick, overhand throw) models. These shorter, latent traits are reliable and show more convergent validity that the full TGMD-3.

### 5.1. Implications for Practitioners

The findings of this study provide some important implications for practitioners. Most importantly, a brief version of the TGMD could be adopted by practitioners for evaluation or investigation based upon the reliability/validity results of the present study. Brief versions of the TGMD are highly desirable due to the lengthy administrative and scoring time required for each assessment (going from 13 to 6 skills should cut time in half). Often administrative time is even lengthier in children with VI due to reduced experience in many motor skills and adapted teaching strategies when necessary [24]. Although saving administrative time is important, it is critical that any version of the TGMD that is adopted should be vetted regarding its psychometric properties. Notably, practitioners should not use a brief version of the TGMD as a normative reference for placement. Rather, the brief versions should be used for program evaluation purposes or to provide additional assessments with the full battery.

### 5.2. Strength, Limitations, and Future Research

To our knowledge, the is the first investigation of brief version of the TGMD-3 for individuals with VI. Along with the innovation behind this inquiry, there are other strengths. Specifically, our analyses incorporated both item difficulty and discrimination. We also considered composite reliability and convergent validity for the latent traits of locomotor and object control skills across multiple models. Although there is always an inherent risk when running multiple models on a single sample, these robust analyses (namely composite reliability and variance extracted) take that risk into account, thus mitigating some of the potential for risk.

In addition to the rigor with our analyses, we specifically focused on practical translations of these findings. Specifically, we suggest that practitioners may now have an efficient way to evaluate formative and summative gains with their students, patients, and stakeholders in a manner that converges with the full battery. Practitioners could use the abbreviated batteries as formative, summative, and criterion references to better evaluate their programs and the extent to which their clients (students, children, patients, etc.) are making gains towards goals. By knowing programmatic effectiveness early and often, assessment data can then better support learning in the long run. Thus, providing ecologically valid brief versions of the TGMD-3 can only support maximation of learning and meeting programmatic goals.

Although this study includes many strengths, it is certainly not without limitations. These limitations should be addressed within future research in the following ways. Further research should be conducted on brief versions of the TGMD for children with VI. Although we conducted several evaluations of various models, we are not suggesting that researchers choose skills at random yet. These data allude to a trend that skill choice may be a possibility; however, further testing and replication are needed with different samples. We suggest that when researchers conduct future inquiry, they select half locomotor and half ball skills in order to maintain the general trait of motor. In addition, researchers using the brief versions of the TGMD should investigate different populations, specifically children with VI outside of camps, both from integrated schools and schools for the blind.

## 6. Conclusions

Scores from all three brief versions of the TGMD tested had acceptable global fit, indicating much potential of the use of a shorter version of the TGMD, saving not only considerable administrative and assessment time, but also potentially providing flexibility to tailor the instrument to be most ecologically valid. Although further research should be conducted, practitioners can adopt a brief version of the TGMD to assess children with VI.

## Figures and Tables

**Table 1:** Descriptive statistics for individual skills.

Gross Motor Skill	Mean	SD	Skewness	Kurtosis	Item Difficulty	Item Discrimination
Locomotor Skills						
Run	5.39	2.53	−0.71	−0.62	0.31	0.60
Gallop	4.06	2.37	−0.47	−0.89	0.05	0.47
Hop	4.16	2.41	−0.07	−0.88	0.13	0.61
Skip	2.97	2.23	−0.22	−1.33	0.17	0.73
Jump	5.06	2.41	−0.43	−0.87	0.23	0.74
Slide	6.51	2.22	−1.72	2.15	0.53	0.51
Object Control Skills						
Two-handed Strike	6.70	2.44	−0.49	−0.42	0.15	0.64
One-handed Strike	3.80	2.60	−0.15	−1.17	0.08	0.37
Dribble	3.99	2.22	−0.70	−1.00	0.42	0.59
Catch	3.33	1.69	0.17	−0.90	0.16	0.67
Kick	4.13	2.34	−0.03	−0.95	0.11	0.50
Overhand Throw	3.19	3.06	0.35	−1.44	0.15	0.68
Underhand Throw	5.21	2.10	−0.92	0.53	0.15	0.42

**Table 2:** Correlations among motor skills.

	Locomotor Skills						Object Control Skills						
	Run	Gallop	Hop	Skip	Jump	Slide	Two-Handed Strike	One-Handed Strike	Dribble	Catch	Kick	Overhand Throw	Underhand Throw
Run	1												
Gallop	0.35	1											
Hop	0.45	0.24	1										
Skip	0.51	0.28	0.45	1									
Jump	0.48	0.27	0.45	0.46	1								
Slide	0.45	0.29	0.36	0.28	0.40	1							
Two-handed Strike	0.49	0.36	0.48	0.55	0.44	0.34	1						
One-handed Strike	0.50	0.30	0.48	0.42	0.42	0.32	0.55	1					
Dribble	0.53	0.27	0.49	0.40	0.45	0.34	0.48	0.53	1				
Catch	0.53	0.27	0.41	0.46	0.43	0.31	0.56	0.49	0.50	1			
Kick	0.57	0.26	0.42	0.39	0.48	0.39	0.54	0.49	0.55	0.47	1		
Overhand Throw	0.50	0.28	0.43	0.47	0.50	0.25	0.50	0.54	0.56	0.57	0.50	1	
Underhand Throw	0.52	0.36	0.30	0.38	0.36	0.46	0.42	0.50	0.41	0.42	0.38	0.44	1

Note. All correlations are significant at *p* < 0.001.

**Table 3:** Fit indices for the measurement model of various versions of the TGMD-3.

	Model chi-Square							
Model	chi-Square	df	***p***	chi/df	CFI	SRMR	RMSEA	RMSEA 90% CI
TGMD-3	79.39	64	0.093	1.24	0.986	0.039	0.028	0.000, 0.047
STAT-6	10.45	8	0.235	1.31	0.994	0.032	0.032	0.000, 0.079
V-6	7.44	8	0.490	0.93	1.000	0.021	0.000	0.000, 0.064
SPT-6	18.07	8	0.021	2.26	0.975	0.033	0.065	0.024, 0.105

**Table 4:** Psychometric properties of the latent traits and motor skills that constitute each model.

Latent Trait	Indicators	Results for Indicators (Skills)			Results for Composites	
		Standardized Loading	Estimate/s.e.	Item Reliability	Composite Reliability	Variance Extracted
TGMD-3						
Locomotor	Run	0.759	23.73 **	0.576	0.79	0.39
	Gallop	0.434	8.32 **	0.188		
	Hop	0.636	15.65 **	0.405		
	Skip	0.660	16.85 **	0.436		
	Jump	0.666	17.08 **	0.443		
	Slide	0.540	11.41 **	0.292		
Object Control	Two-handed Strike	0.741	19.44 **	0.549	0.87	0.50
	One-handed Strike	0.721	17.90 **	0.520		
	Dribble	0.714	17.47 **	0.510		
	Catch	0.708	17.01 **	0.501		
	Kick	0.701	16.63 **	0.492		
	Overhand Throw	0.730	18.52 **	0.533		
	Underhand Throw	0.615	12.20 **	0.379		
STAT-6						
Locomotor	Run	0.751	17.10 **	0.563	0.70	0.42
	Hop	0.634	12.93 **	0.401		
	Slide	0.546	10.49 **	0.298		
Object Control	One-handed Strike	0.733	16.10 **	0.537	0.79	0.55
	Dribble	0.762	17.72 **	0.580		
	Overhand Throw	0.730	15.91 **	0.534		
V-6						
Locomotor	Run	0.727	15.93 **	0.529	0.63	0.37
	Gallop	0.438	7.78 **	0.192		
	Hop	0.618	12.84 **	0.381		
Object Control	Two-handed Strike	0.746	17.00 **	0.556	0.77	0.53
	Kick	0.733	16.24 **	0.537		
	Overhand Throw	0.697	14.51 **	0.485		
SPT-6						
Locomotor	Run	0.736	18.34 **	0.542	0.72	0.47
	Hop	0.628	13.72 **	0.394		
	Skip	0.680	15.74 **	0.463		
Object Control	Two-handed Strike	0.755	16.62 **	0.570	0.72	0.46
	Kick	0.686	13.81 **	0.471		
	Underhand Throw	0.582	10.15 **	0.339		

Note. ** denotes *p* < 0.001.

## Data Availability

The data presented in this study are available on request from the corresponding author.
